# *Xylella fastidiosa* modulates exopolysaccharide polymer length and the dynamics of biofilm development with a β-1,4-endoglucanase

**DOI:** 10.1128/mbio.01395-23

**Published:** 2023-10-13

**Authors:** Claudia Castro, Ikenna Ndukwe, Christian Heiss, Ian Black, Brian M. Ingel, Matthew Guevara, Yuling Sun, Parastoo Azadi, Qiang Sun, M. Caroline Roper

**Affiliations:** 1 Department of Microbiology and Plant Pathology, University of California, Riverside, California, USA; 2 Complex Carbohydrate Research Center, University of Georgia, Athens, USA; 3 Department of Computer Science, Wellesley College, Wellesley, Massachusetts, USA; 4 Department of Biology, University of Wisconsin, Stevens Point, Wisconsin, USA; University of Nebraska-Lincoln, Lincoln, Nebraska, USA

**Keywords:** biofilms, exopolysaccharide, grapevine, plant pathogens

## Abstract

*Xylella fastidiosa* is a Gram-negative bacterium that causes disease in many economically important crops. It colonizes the plant host xylem and the mouthparts of its insect vectors where it produces exopolysaccharide (EPS) and forms robust biofilms. Typically, the ability to form a biofilm enhances virulence, but *X. fastidiosa* does not fit neatly into that paradigm. Instead, *X. fastidiosa* enters into biofilms to attenuate its movement in the xylem, which, in turn, slows disease progression. In most of its over 600 known plant hosts, *X. fastidiosa* behaves as a benign commensal, but in some hosts like *Vitis vinifera* grapevines, it acts as a pathogen. Its ability to attenuate its own virulence in susceptible hosts may be a remnant of its commensal lifestyle in other hosts. Here, we demonstrate that *X. fastidiosa* utilizes a β-1,4 endoglucanase to cleave its self-produced β-1,4-glucan exopolysaccharide polymer to process it from a higher molecular weight to a lower molecular weight polymer. This processing mediates surface adherence of the cells and ultimately governs overall biofilm architecture, indicating enzymatic pruning of the EPS plays a key role in biofilm-mediated attenuation of *X. fastidiosa in planta* and, thus, is a key vestige that links its commensal behaviors to its parasitic behaviors in specific hosts.

**IMPORTANCE:**

It is well established that exopolysaccharide (EPS) is an integral structural component of bacterial biofilms necessary for assembly and maintenance of the three-dimensional architecture of the biofilm. However, the process and role of EPS turnover within a developing biofilm is not fully understood. Here, we demonstrated that *Xylella fastidiosa* uses a self-produced endoglucanase to enzymatically process its own EPS to modulate EPS polymer length. This enzymatic processing of EPS dictates the early stages of *X. fastidiosa*’s biofilm development, which, in turn, affects its behavior *in planta*. A deletion mutant that cannot produce the endoglucanase was hypervirulent, thereby linking enzymatic processing of EPS to attenuation of virulence in symptomatic hosts, which may be a vestige of *X. fastidiosa*’s commensal behavior in many of its other non-symptomatic hosts.

## INTRODUCTION

*Xylella fastidiosa* is a fastidious, Gram-negative bacterium that causes disease in many economically important crops including grape, olive, citrus, coffee, and almond. This plant-pathogenic bacterium resides exclusively in the xylem of its plant hosts and in the foregut of its xylem-feeding hemipteran insect vectors, such as the sharpshooters, *Graphocephala atropunctata* and *Homalodisca vitripennis*, and the spittlebug, *Philaenus spumarius,* among others (1, 2). *X. fastidiosa* is endemic to the Americas and emerged in Europe in 2013 where it is causes olive quick decline disease and is killing a significant number of olive trees in Italy (3). *X. fastidiosa* subsp. *fastidiosa*, the causal agent of Pierce’s disease of grapevine (PD), was first observed in southern California in the 1800s and remains a significant problem for raisin, table, and wine grape growers in the United States.

*In planta*, attachment to the xylem wall is a critical part of the infection and insect acquisition processes. However, long-range systemic movement in the xylem tissue requires that the bacteria be in a non-adherent, exploratory state, predominately comprised of planktonic cells (4). Thus, *X. fastidiosa* must maintain subsets of its population that displays these two disparate (non-adhesive vs adhesive) cellular phenotypes to achieve systemic colonization throughout the xylem network and also be amenable for insect acquisition, which requires that the bacterium adheres to the insect cuticle. Proteinaceous surface adhesins as well as carbohydrate-based adhesins like lipopolysaccharide and exopolysaccharide (EPS) mediate attachment to plant and insect surfaces (5 – 7).

Typically, the ability to attach to host tissues and associate into biofilms promotes virulence. However, *X. fastidiosa* does not fit neatly into that paradigm. Mutants that are defective in surface attachment and/or cell-cell aggregation are predominantly hypervirulent in grapes indicating that having a population skewed toward non-adherent, planktonic cells enhances virulence, rather than diminishing it (3, 8 – 10). Thus, it is speculated that *X. fastidiosa* attenuates its virulence through auto-aggregation and adherence to the xylem wall of grapevines to temper its systemic movement to avoid killing its plant host too quickly (11). This may also explain why such a long latent period (weeks to months) occurs between initial infection and symptom development. This self-limiting behavior may be a remnant of its commensal lifestyle in the majority of its other non-symptomatic plant hosts.

Biofilms are organized multicellular aggregates encased in a matrix (commonly self-produced) that protects against environmental stresses and plays a role in community ecology by promoting signaling and communication among biofilm inhabitants. Entering into the biofilm lifestyle begins with the attachment of planktonic cells to a surface and is mediated by fimbrial and afimbrial adhesins among other cell surface entities that contribute to overall surface charge. After attachment, cells multiply and auto-aggregate to form microcolonies. Microcolonies develop into macrocolonies, and biofilm-associated cells produce extracellular polymeric substances such as polysaccharides, proteins, extracellular DNA, and lipids that form the biofilm matrix. Once the biofilm is mature, it can be reconfigured and/or degraded to promote growth of the biofilm or to initiate cell dispersal from the biofilm. Cell dispersal from the biofilm enables cells to return to the planktonic state to initiate exploration and the colonization of a new niche (11, 12).

The glycoside hydrolase, EngXCA2, is a β-1,4-endoglucanase (EGase) with a hydrolytic N-terminal domain and a C-terminal cellulose binding domain (13). This protein belongs to the glycoside hydrolase family 5 and can degrade carboxymethyl cellulose and xyloglucan *in vitro*. Moreover, in tandem with a polygalacturonase, EngXCA2 digests grapevine pit membranes (14, 15). Glycoside hydrolases from this family can also cleave bacterial carbohydrate polymers, and many of them harbor catalytic promiscuity to enable cleavage of more than one substrate (16). Interestingly, a *X. fastidiosa ΔengXCA2* deletion mutant is hypervirulent *in planta* suggesting a secondary virulence mechanism for this EGase in addition to its role in dismantling pit membranes (13). The composition of the EPS was predicted to consist of a tetrasaccharide subunit with a β-1,4-glucan structurally similar to, but not exactly like, xanthan gum (17). Our compositional and structural analyses indicated that, indeed, *X. fastidiosa* wild-type EPS is similar to xanthan, but with notable differences (18). Specifically, *X. fastidiosa* EPS consists of octasaccharide repeating units composed of a β-1,4-glucan backbone with two different three-linked side chains, one with terminal β-glucuronic acid and α-1,2-mannose residues (60%) and the other with terminal β-glucose and α-1,2-mannose residues (40%). We did not detect any acetylation on the EPS side chain as predicted by the genomic analysis (18). Because *X. fastidiosa* EPS consists of a β-1,4-glucan backbone, we posited that EngXCA2 can also utilize the EPS backbone as a substrate.

Because the Δ*engXCA2* mutant had a strikingly hypermucoid phenotype *in vitro*, we tested the hypothesis that EngXCA2 modulates EPS processing through enzymatic degradation of the β-1,4-glucan backbone of its EPS that has downstream impacts on cellular and biofilm physiology. Indeed, we demonstrated that EngXCA2 can digest the EPS polymer and that lack of enzymatic processing by EngXCA2 affects polymer length and accumulation of EPS on the cell surface. Deletion of *engXCA2* also had severe impacts on biofilm development and overall architecture both *in vitro* and *in vivo* indicating that enzymatic processing of EPS by an endogenous glycoside hydrolase is a key mechanistic interaction of the microbe-microbe interactions within the developing biofilm and also during the host-microbe interaction that dictates where this bacterium falls on the spectrum of symbioses that range from parasitism to commensalism.

## RESULTS

### Δ*engXCA2* is hypermucoid *in vitro*

**Fig 1:**
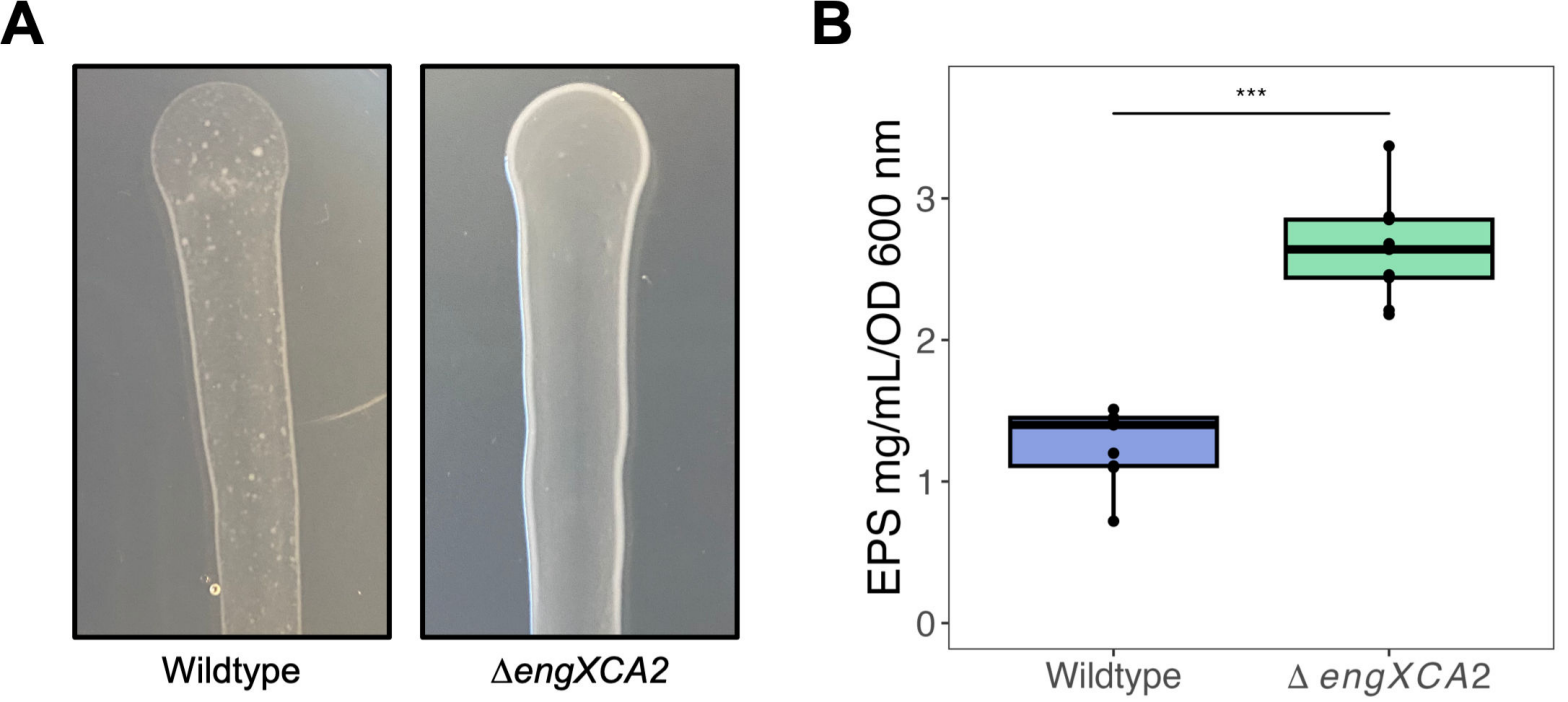
Δ*engXCA2* produces significantly more exopolysaccharide (EPS) *in vitro.* (**A**) Representative photographs of Δ*engXCA2* and wild-type growth on PD3 solid medium. Δ*engXCA2* did not aggregate as compared to wild type and has a glistening shiny surface. (**B**) Quantification of EPS production in wild-type and Δ*engXCA2* cells propagated in liquid culture indicated that Δ*engXCA2* produces more EPS/mL than wild-type *X. fastidiosa* (*n* = 9, analysis of variance [ANOVA] followed by Tukey’s honest significant difference test).

Qualitatively**,** Δ*engXCA2* had a hypermucoid phenotype with a visible slime layer when propagated on PD3 medium as compared to wild-type *X. fastidiosa* (Fig. 1A). Quantitatively, Δ*engXCA2* cells produced significantly more EPS when propagated in liquid *Xylella fastidiosa* medium (XFM) medium supplemented with pectin as compared to wild type (Fig. 1B).

### Recombinant EngXCA2 cleaves *X. fastidiosa* EPS

**Fig 2:**
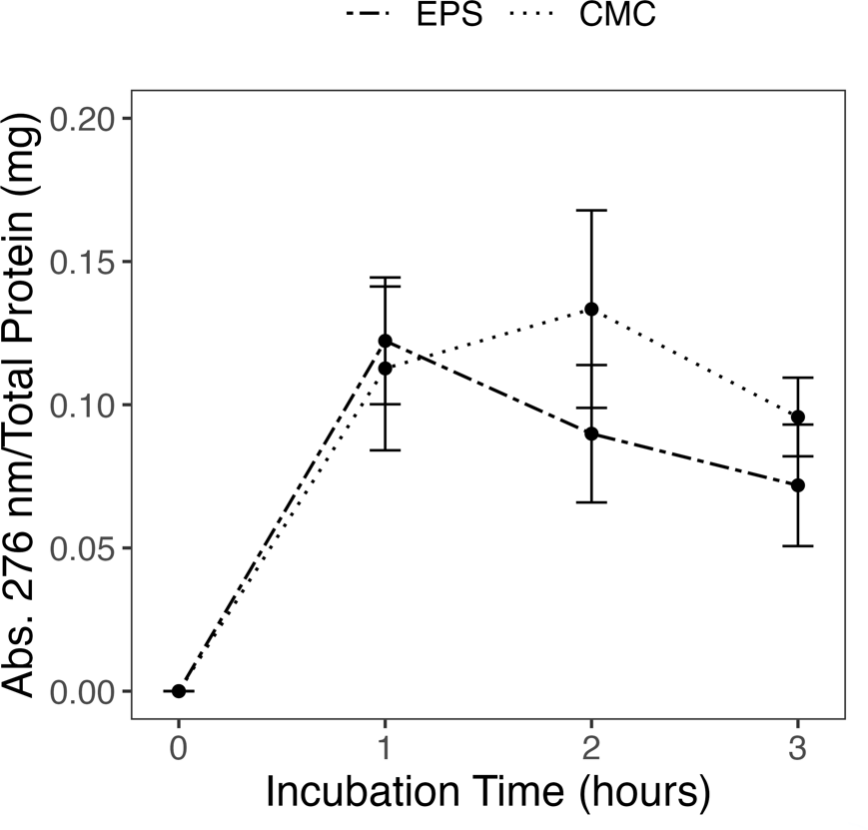
Recombinant *X. fastidiosa* EngXCA2 cleaves Δ*engXCA2* exopolysaccharide. *E. coli* BL21 (DE3) lysates containing recombinant *X. fastidiosa* EngXCA2 were incubated with Δ*engXCA2 X. fastidiosa* EPS or CMC as a positive control. Lysates from *E. coli* carrying pET-20(b)+ empty vector incubated with EPS or CMC were used as negative controls. Data presented were normalized to the respective negative control and represent the average of three independent experiments each with three replicates (*n* = 9). Error bars represent standard error.

Because Δ*engXCA2* had a hypermucoid phenotype that affected biofilm development, we hypothesized that EngXCA2 modulates EPS turnover and/or biosynthesis through enzymatic degradation of the β-1,4-glucan backbone of EPS. We performed a reducing sugar assay to test EPS as a potential substrate for recombinant *X. fastidiosa* EngXCA2 protein expressed in *Escherichia coli* (15). Recombinant EngXCA2 can cleave carboxymethylcellulose (CMC) and xyloglucan *in vitro*, and here, we used CMC as a positive control in the reducing sugar assay (15). Indeed, recombinant EngXCA2 cleaved EPS from Δ*engXCA2* as indicated by the production of reducing ends over time (Fig. 2). These data confirm that EngXCA2 can utilize EPS as a substrate, supporting our hypothesis that EngXCA2 plays a role in maintenance of the biofilm EPS matrix by enzymatic processing of the EPS polymer.

### Δ*engXCA2* EPS has a higher molecular weight than wild-type EPS

**Fig 3:**
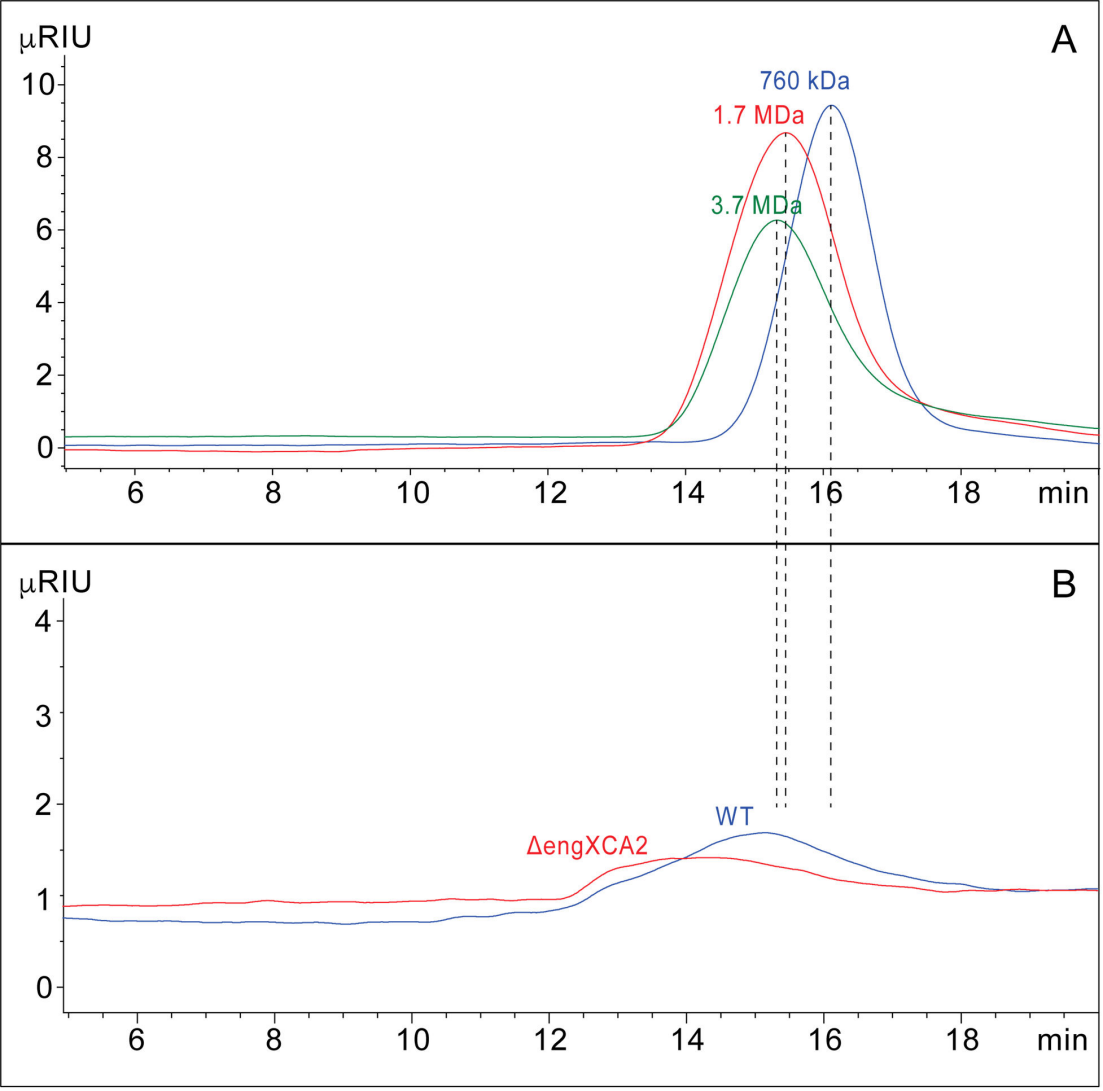
Size-exclusion chromatography of *X. fastidiosa* wild-type and Δ*engXCA2* EPSs. Wild-type and Δ*engXCA2* EPSs (**B**) were compared to dextran standards (**A**) and have a molecular weight of >3.7 MDa. Δ*engXCA2* EPS (red) eluted earlier than the wild-type EPS (blue) indicating it is larger in size than wild-type EPS. Vertical lines indicate the peak maximum of the dextran standards (0.76, 1.7, and 3.7 kDa).

**Fig 4:**
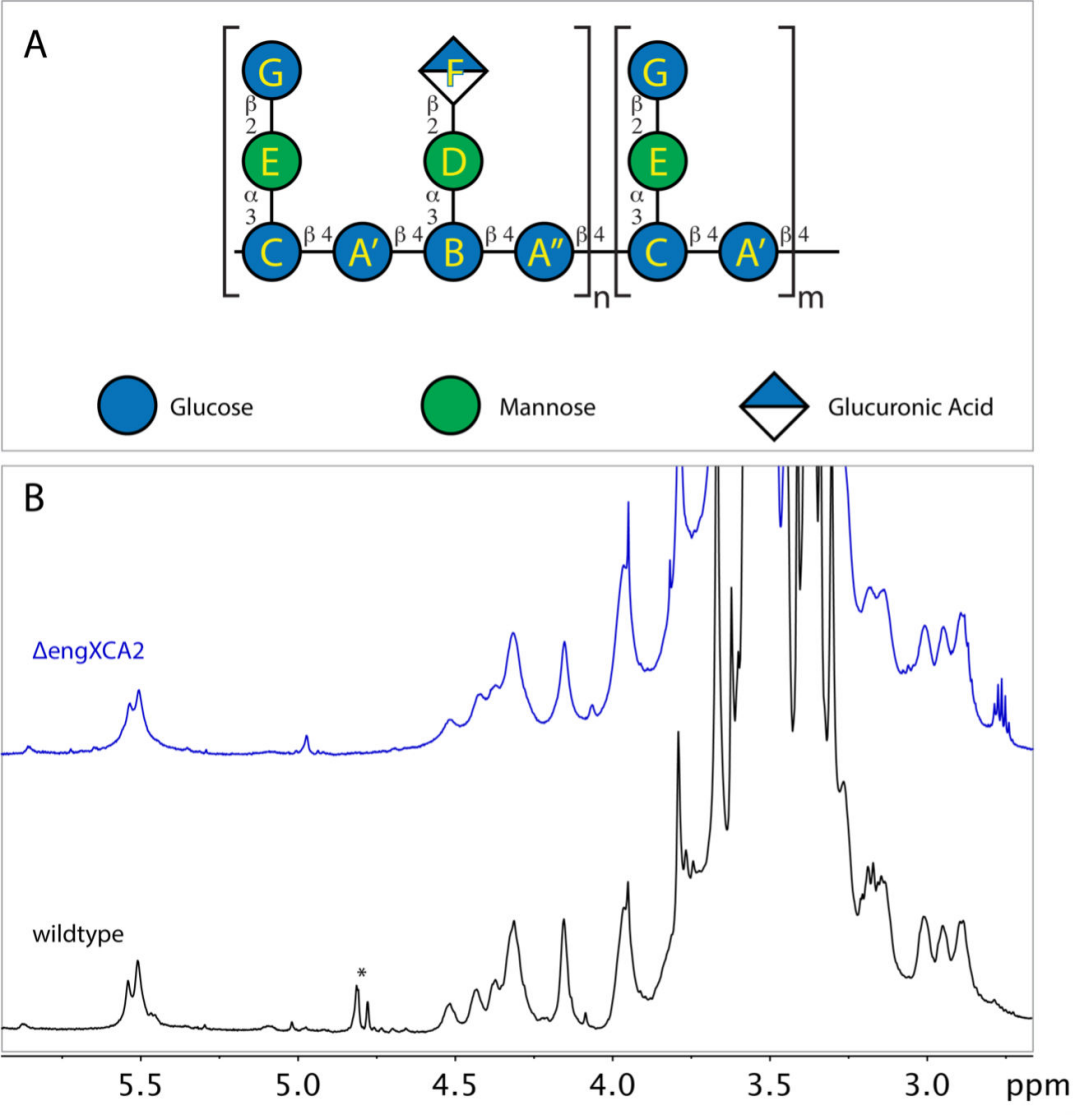
Structure of *X. fastidiosa* exopolysaccharide (EPS). (**A**) *X. fastidiosa* wild-type and Δ*engXCA2* EPS were identical in sequence and composed of a β-1,4-glucan backbone with two different three-linked side chains, one with terminal β-glucuronic acid and α-1,2-mannose residues and the other with terminal β-glucose and α-1,2-mannose residues. About 60% of the side chains terminated in β-Glc and 40% in β-GlcA residues. (**B**) ^1^H NMR spectra of permethylated *X. fastidiosa* and *ΔengXCA2* EPSs indicate that the subunits for both *X. fastidiosa* and *ΔengXCA2* were similar. The set of peaks labeled with an asterisk corresponds to some monosaccharides that were released by mild acid hydrolysis.

We hypothesized that lack of EngXCA2 processing of the EPS in the Δ*engXCA2* mutant resulted in a larger molecular weight EPS molecule produced by Δ*engXCA2* compared to wild-type cells that, in turn, is responsible for the hypermucoidy that we observed in the Δ*engXCA2* mutant. We performed size-exclusion chromatography (SEC) to determine the molecular weight of the polymers from both strains. Our analysis showed that the mutant EPS eluted earlier at 14.4 min, compared to the wild-type EPS at 15.2 min, confirming that the Δ*engXCA2* mutant EPS is of a larger molecular weight than the wild-type EPS. However, both EPSs were larger than several of the dextran standards that we used, which prevented us from determining their exact size, which is at least 3.75 MDa (Fig. 3). *X. fastidiosa* EPS consists of octasaccharide repeating units composed of a β-1,4-glucan backbone with two different three-linked side chains, one with terminal β-glucuronic acid and α-1,2-mannose residues (60%) and the other with terminal β-glucose and α-1,2-mannose residues (40%) (Fig. 4A). Nuclear magnetic resonance (NMR) data indicated that both EPS subunits from wild-type and Δ*engXCA2* were identical indicating that EngXCA2 does not play a role in altering subunit composition (Fig. 4B).

### Δ*engXCA2* is impaired in the early steps of biofilm formation: cell-surface attachment and cell-cell aggregation

**Fig 5:**
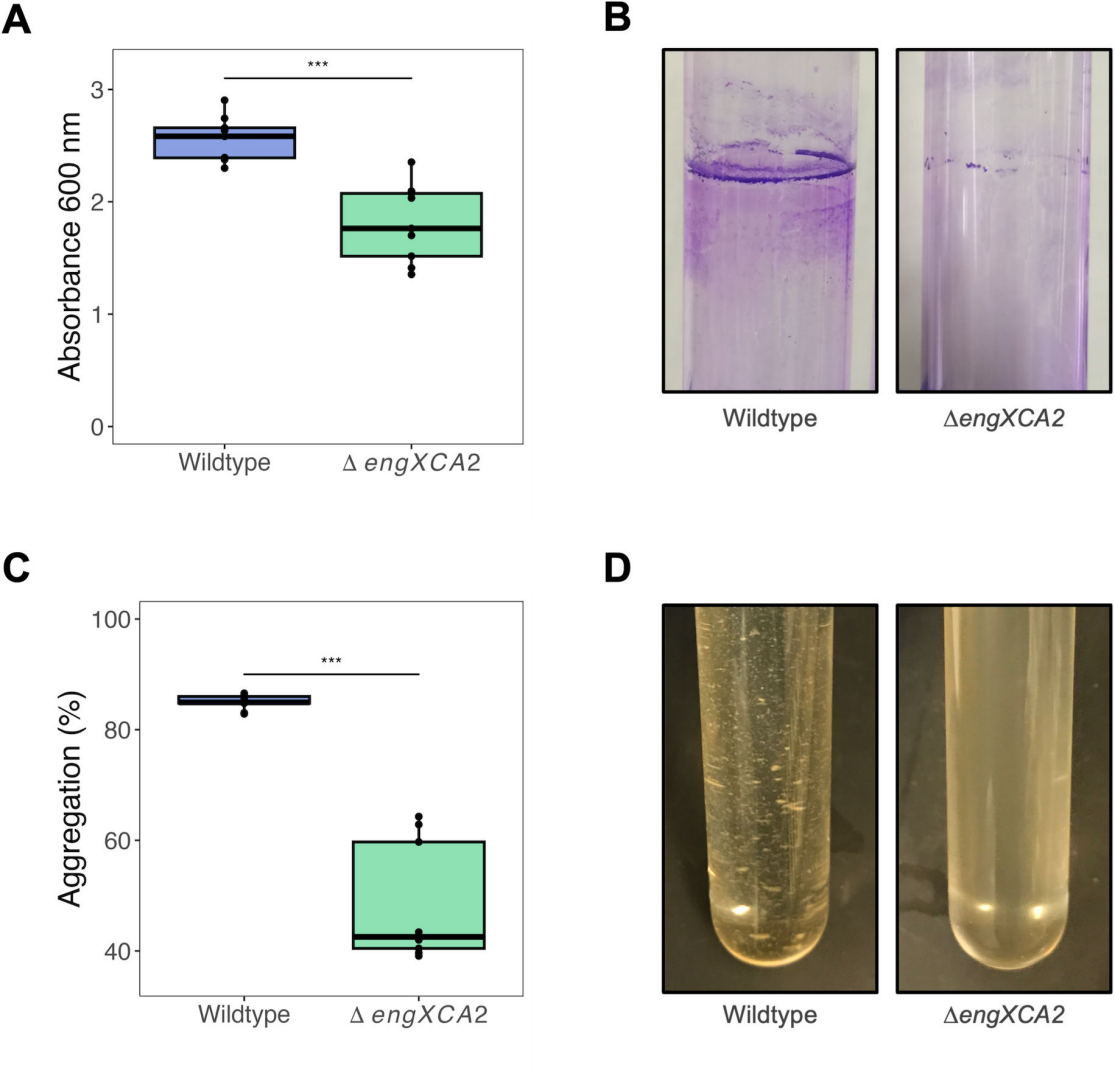
Δ*engXCA2* was impaired in cell-cell aggregation and cell-surface attachment. (**A**) Quantification of cell-glass surface attachment at the air-liquid interphase (*n* = 9, ANOVA followed by Tukey’s Honest Significant Difference test; asterisks indicate level of significance: *P < 0.05, **P < 0.01, ***P < 0.001] indicated that Δ*engXCA2* attached significantly less to a glass surface than wild type. (**B**) Representative images of crystal violet stained biofilm cells attached to glass at the air-liquid interphase. (**C**) Cell-cell aggregation percentage of wild type and Δ*engXCA2* propagated in liquid medium indicating reduced aggregation in liquid culture for Δ*engXCA2* (*n* = 9, ANOVA followed by Tukey’s Honest Significant Difference test; asterisks indicate level of significance: *P < 0.05, **P < 0.01, ***P < 0.001). (**D**) Representative images of wild-type cell aggregates and Δ*engXCA2* cells demonstrating reduced aggregation in liquid culture for Δ*engXCA2*.

When grown upright in glass tubes in liquid medium, *X. fastidiosa* forms biofilms at the air-liquid interface. A critical first step in this process is cell-surface attachment. We quantified cell-surface attachment using glass as the surface substrate coupled with a colorimetric crystal violet assay. Wild-type biofilms formed a thicker attachment ring on the tube surface at the air-liquid interface and, thus, retained more crystal violet than Δ*engXCA2* biofilms indicating that Δ*engXCA2* was significantly impaired in attachment to the glass substrate (Fig. 5A and B). Cell-cell aggregation or auto-aggregation is another critical early step in forming a biofilm (19, 20). *X. fastidiosa* is particularly adept at auto-aggregation and forms large, visible aggregates in liquid medium, which are very difficult to disperse via agitation or vortexing. Quantitatively, Δ*engXCA2* aggregated significantly less than wild type (Fig. 5C). In addition, this lack of aggregation phenotype was visually observed because Δ*engXCA2* appears turbid in liquid culture rather than in aggregates like the wild-type parent (Fig. 5D).

### Δ*engXCA2 in vitro* biofilms have altered three-dimensional biofilm architecture

**Fig 6:**
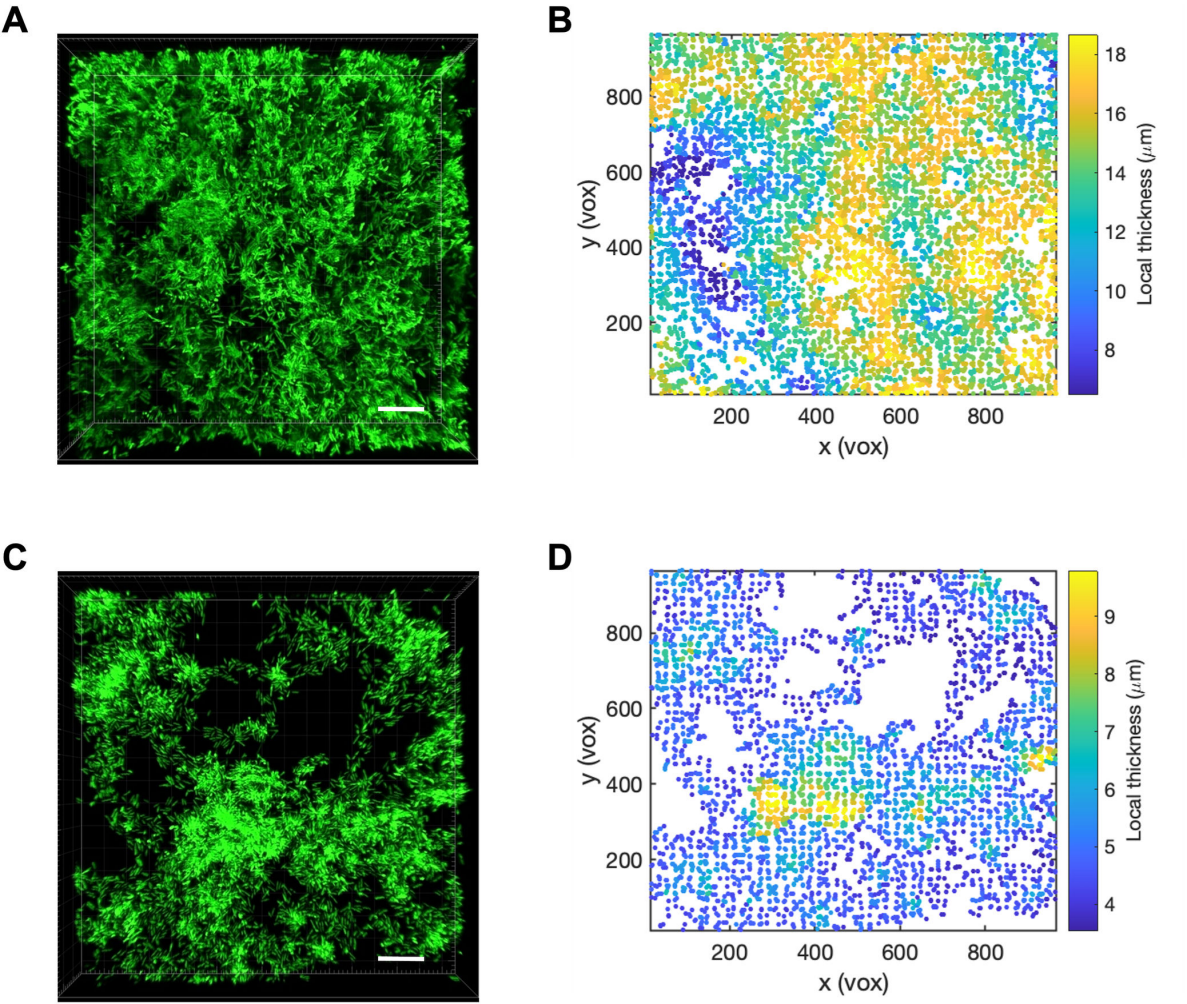
Δ*engXCA2 in vitro* biofilms were structurally impaired compared to wild type. Representative confocal laser scanning microscopy images of (**A**) wild-type and (**C**) Δ*engXCA2 in vitro* biofilms stained with SYTO 9 green. Scale bar, 10 µm. Localized thickness (micrometers) landscape of (**B**) wild-type and (**D**) Δ*engXCA2 in vitro* biofilms. Vox, voxel.

**Fig 7:**
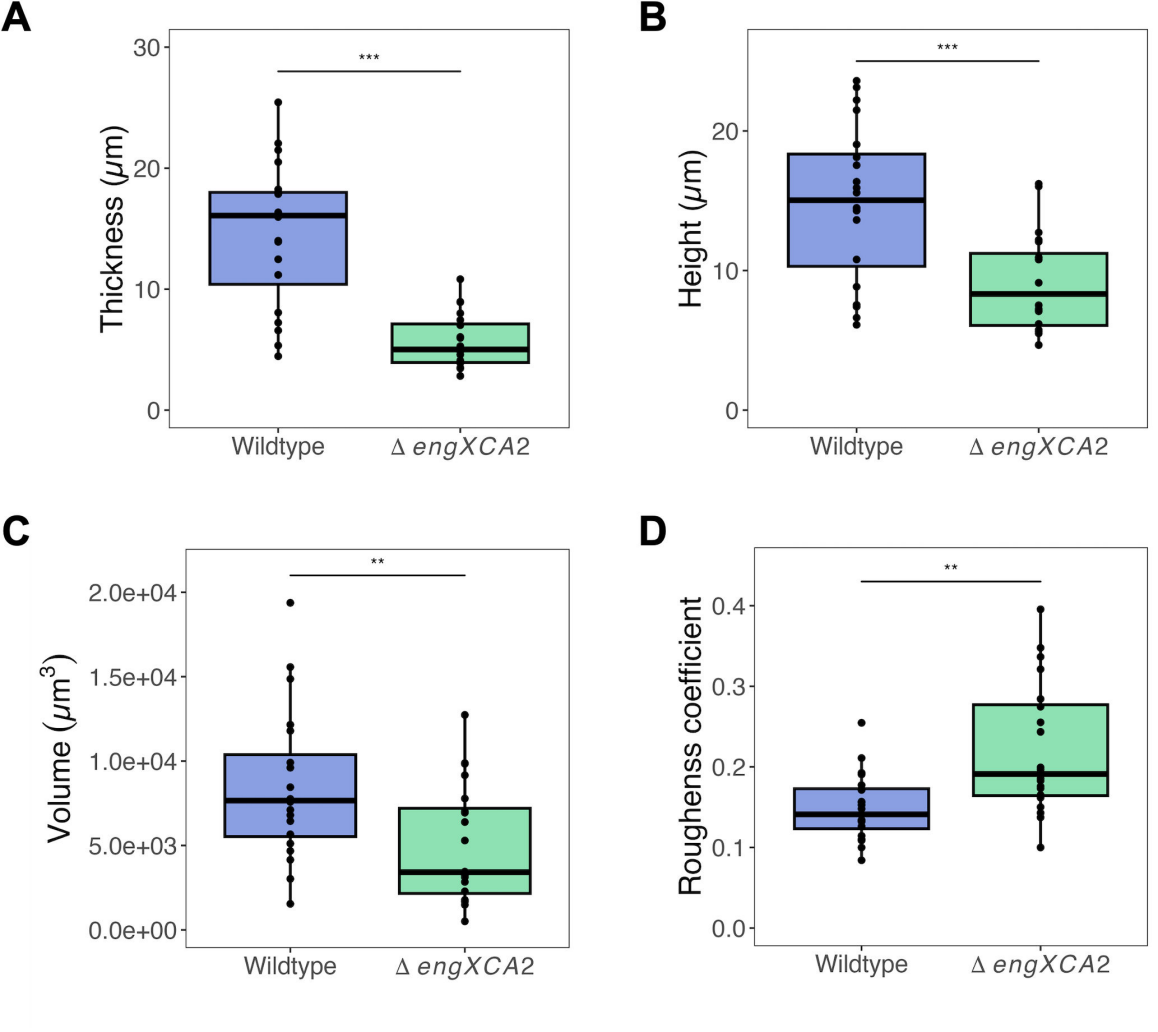
Δ*engXCA2 in vitro* biofilms were thinner and had lower biomass and a higher roughness coefficient. (**A**) Average thickness, (**B**) height (highest peak), (**C**) average volume, and (**D**) roughness coefficient for wild-type and *ΔengXCA2 in vitro* biofilms (*n* = 25, ANOVA followed by Tukey’s honest significant difference test; asterisks indicate level of significance: *P < 0.05, **P < 0.01, ***P < 0.001). Δ*engXCA2* had significantly less biomass with an average of 5.02 mm^3^ in comparison to wild type that had an average biomass volume of 8.47 mm^3^. Average roughness coefficients for the two strains indicated that Δ*engXCA2* biofilms had a more uneven or rougher surface than wild type which had a smoother surface, 0.221 and 0.149, respectively.

To determine how overproduction of EPS affected Δ*engXCA2* biofilm architecture, we examined Δ*engXCA2* biofilms grown on a glass surface using confocal laser scanning microscopy. After 4 days of incubation in liquid medium, both wild-type and Δ*engXCA2* cells had formed biofilms on the glass slide. However, Δ*engXCA2* biofilms colonized less glass surface area than wild type, and localized thickness measurements obtained using BiofilmQ software indicated that Δ*engXCA2* biofilms were thinner than wild-type biofilms (21). Specifically, Δ*engXCA2* and wild-type biofilms were 5.68 µm and 14.58 µm in thickness, respectively (Fig. 6A through D and 7A). Furthermore, we used BiofilmQ software to segment the z-stack images and measure each point from the glass substrate to the highest point of the biofilm (Fig. 6B and D). From this analysis, we determined that the highest peaks in the wild-type biofilms had a median height of 15.03 µm and the highest peaks in the Δ*engXCA2* biofilms had a median height of 8.32 µm (Fig. 7B). Overall, Δ*engXCA2* had significantly less biomass with an average of 5.02 mm^3^ in comparison to wild type that had an average biomass volume of 8.47 mm^3^ (Fig. 7C). Finally, average roughness coefficients for the two strains indicated that Δ*engXCA2* biofilms had a more uneven or rougher surface than wild type which had a smoother surface, 0.221 and 0.149, respectively (Fig. 7D). Taken together, these assay results show that Δ*engXCA2* cells were compromised in their capacity to construct a mature biofilm architecture typical of wild-type *X. fastidiosa*.

### *In vivo, ΔengXCA2* biofilms were encased in copious amounts of EPS

**Fig 8:**
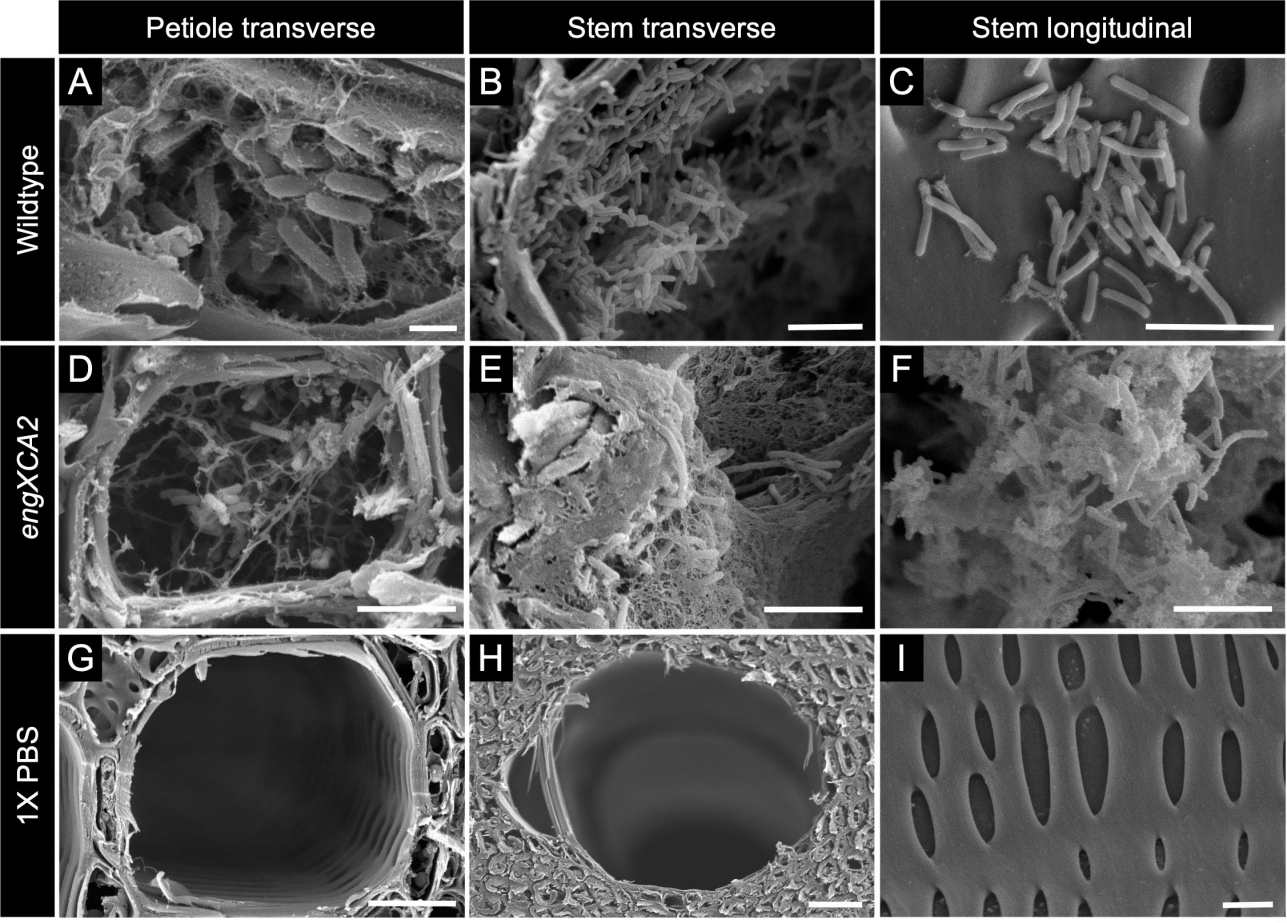
Δ*engXCA2* biofilms were associated with larger amounts of EPS in the xylem vessels of *Vitis vinifera* ‘Cabernet Sauvignon stems and petioles. Scanning electron micrographs of vessels in the vines inoculated with (**A–C**) wild type, (**D–F**) Δ*engXCA2*, and (**G–I**) 1× PBS. First column from the left, petiole transverse sections. Second column, stem transverse sections. Third column, stem longitudinal sections. (**A–C**) *X. fastidiosa* wild-type biofilms were associated with varying amounts of EPS, and individual bacterial cells were clearly visible in the vessel lumen. (**D–F**) Δ*engXCA2* cells developed into biofilms and were encased in large amounts of EPS in the vessel lumen. (**G–H**) Empty xylem vessels of PBS-inoculated vines were free of bacterial cells and biofilms. (**I**) Secondary xylem pit membranes were intact in PBS-inoculated vines. Scale bar is 1 µm in panel **A**, 5 µm in panels B–F and I, 10 µm in panel **G**, and 30 µm in panel **H**.

Because Δ*engXCA2* was hypermucoid and impaired in biofilm formation *in vitro*, we investigated if these phenotypes were recapitulated *in vivo*. Using scanning electron microscopy (SEM), we analyzed Δ*engXCA2* and wild-type biofilms that were formed in the xylem vessels of stems and petioles of grapevines inoculated with wild type (Fig. 8A through C), Δ*engXCA2* (Fig. 8D through F), or 1× phosphate-buffered saline (PBS) (Fig. 8G through I) and revealed some differences in the formation and morphology of biofilms in both stem and petioles by the two bacterial genotypes. In stems, wild-type cells colonizing vessels were observed as single cells attached to the vessel lateral walls (Fig. 8B and C), as microcolonies, and as part of mature biofilms with EPS that was tightly associated with the biofilm. In contrast, Δ*engXCA2* colonizing vessels in stems was frequently present as dense clusters of cells encased in fibrous, thick EPS that filled the vessel lumen and was not frequently observed as individual cells or microcolonies (Fig. 8E through F). In petioles, the majority of Δ*engXCA2* cells were part of biofilms that extensively colonized the petiole vessels (Fig. 8D), whereas wild-type cells were rarely observed in petiole samples and if they were observed they occurred mostly singly (Fig. 8A). No bacterial cells were observed in vessels of either stems or petioles of 1× PBS-inoculated vines (Fig. 8G through I).

## DISCUSSION

It is well established that EPS is a major component of the extracellular matrix and is critical for building and maintaining the architecture of bacterial biofilms (12). However, the mechanisms that underlie EPS processing and turnover within a developing biofilm are not well understood. *X. fastidiosa* EPS is made up of octasaccharide repeating units with a β-1,4-glucan backbone with two different alternating three-linked side chains, one with terminal β-glucuronic acid and α-1,2-mannose residues and the other with terminal β-glucose and α-1,2-mannose residues. EngXCA2 digests the EPS polymer *in vitro*, and the EPS subunits from wild type and Δ*engXCA2* were identical indicating that EngXCA2 likely does not play a role in altering subunit composition but rather serves to cleave the glucan backbone to reduce polymer length. Similarly, the human bacterial pathogen, *Pseudomonas aeruginosa* produces a glycoside hydrolase, PelA, that is involved in the production of a lower molecular weight form of its Pel glycopolymer (22).

Lack of EngXCA2-mediated enzymatic digestion of the EPS led to a notable production of EPS and a hypermucoid phenotype. Specifically, the Δ*engXCA2* colonies had a glistening mucoid surface, whereas wild-type colonies had a smooth dull surface. We speculate that the differences in colony morphology to accumulation of a higher molecular weight EPS on the cell surface due to incomplete processing of the EPS by the Δ*engXCA2* mutant that lacks enzymatic modification of the EPS by EngXCA2. Wild-type *X. fastidiosa* EPS is primarily tightly associated with the bacterial cell surface, and our data support our hypothesis that *X. fastidiosa* utilizes EngXCA2 to enzymatically cleave and maintain an EPS layer that is tightly associated with the cells and biofilm (23). In the *P. aeruginosa* system, PelA enzymatic activity also reduces PelA polymer length, but rather than mediating formation of a tightly associated capsule, PelA activity shifts the Pel glycopolymer from being primarily cell-associated to a loose, secreted polymer (22).

It is not clear if *X. fastidiosa* EPS processing occurs extracellularly or in the periplasmic space prior to secretion out of the cell. However, following repeated attempts, EngXCA2 has not been detected in the *X. fastidiosa* secretome suggesting it is localized internally or is transiently present in the secretome (24, 25). In other systems, like *P. aeruginosa*, overexpression of the periplasmic glycoside hydrolase, PslG, affects Psl EPS synthesis, and the alginate lyase, AlgL, is also localized in the periplasm (26 – 28). Further localization studies for *X. fastidiosa* EngXCA2 are warranted to determine if it is the part of the extracellular secretome or localized to the cell surface, periplasm, or cytoplasm.

Dissolution is considered to be the last stage of biofilm development when communal cells leave the biofilm and enter the planktonic state to colonize a new niche and glycoside hydrolases have been implicated in dissolution (11, 12). However, little is known about the mechanistic role of endogenous EPS-targeting enzymes in the early stages of biofilm development, such as cell-surface attachment and microcolony/macrocolony formation and how their modulation of EPS affects biofilm architecture. Interestingly, in the *X. fastidiosa* system, EngXCA2-mediated EPS processing played an intrinsic role in anchoring of cells to the surface and to each other during biofilm initiation. Over the course of development of the biofilm, Δ*engXCA2* biofilms had significantly lower volume and thickness as compared to wild-type biofilms indicating that production of a higher molecular weight EPS also impeded comprehensive spatial biofilm morphology. We speculate that EPS accumulation blocks cell surface adhesins required for cell-cell and cell-surface adhesion. The concomitant impairment of aggregative and adhesion phenotypes of Δ*engXCA2* corroborates this hypothesis.

Immunological studies coupled with confocal microscopy of wild-type *X. fastidiosa* biofilms indicate that the β-1,4-linked glucan EPS is intercalated throughout the mature biofilm matrix and is tightly associated with the bacteria (23). In vessels of grapevine stem, scanning electron micrographs indicated that wild-type biofilms were encased in variable amounts of a stringy EPS matrix with individual bacterial cells clearly visible. In contrast, Δ*engXCA2* biofilms were encased in copious amounts of a fibrous, ropy EPS, and individual bacterial cells were largely obscured by matrix. Moreover, Δ*engXCA2* biofilms in stem vessels were more compacted and filled a larger area of the vessel lumen in comparison to wildtype. Both wild-type and Δ*engXCA2* cells were visible in petiole vessels, but Δ*engXCA2* was present in large aggregates and wild type was only sparsely present as individual cells. This suggests that Δ*engXCA2* moved more readily than wild type into the petiole from the stems where it was initially inoculated. Systemic movement within the xylem network is directly linked to virulence, and *X. fastidiosa* mutants that can more readily move through the xylem network are generally hypervirulent (8 – 10). In a previous study, we demonstrated that Δ*engXCA2* was hypervirulent in *Vitis vinifera*. Specifically, vines inoculated with Δ*engXCA2* developed disease at a faster rate and displayed more severe symptoms than those inoculated with the wild-type parental strain (13). We speculate that absence of EngXCA2-mediated EPS processing skews Δ*engXCA2* toward a planktonic, exploratory state that enables the Δ*engXCA2* to more rapidly colonize the xylem network because of its aggregation and adhesion deficient phenotypes, which leads to hypervirulence.

Much is known about the regulatory components that govern transcriptional regulation of EPS synthesis to ensure production occurs within the correct environmental and communal context. Even small perturbations in these regulatory cascades can have severe consequences for overall biofilm construction and architecture (2, 29 – 32). Here, we show that enzymatic processing of the EPS is an additional facet that controls the association of EPS matrix with the cells, which is critical to biofilm initiation. *X. fastidiosa* enters into biofilm formation to attenuate rather than enhance its virulence in susceptible grapevines (8 – 10). It is speculated that abating systemic movement via biofilm association with the xylem wall may be a remnant of its commensal behavior in most of its plant hosts. Understanding how commensalism can “go wrong” and manifest into pathologies in specific hosts but not others is a useful vantage point from which to study the determinants of virulence and pathogenicity.

## MATERIALS AND METHODS

### Bacterial strains

*X. fastidiosa* subsp. *fastidiosa* Temecula1 wild-type and Δ*engXCA2* mutant strains were grown on solid PD3 medium at 28°C for 5 days. In brief, the Δ*engXCA2* mutant was obtained by homologous recombination between flanking regions of the deletion construct and the same regions within the *X. fastidiosa* chromosome to facilitate complete removal of the target gene and subsequent insertion of the antibiotic resistance gene as described in reference 13. Repeated attempts to obtain transformant colonies for gene complementation of Δ*engXCA2* were previously unsuccessful and again during this study (13). Because *X. fastidiosa* complementation tools are limited, attempts at gene complementation are often not successful (33).

### Exopolysaccharide extraction and quantification

*X. fastidiosa* cells were harvested from solid PD3 plates and adjusted to OD_600_ of 0.5 in XFM medium. Cells were added to 15-mL XFM-pectin liquid cultures to a final OD_600_ of 0.05 and grown at 28°C, 180 rpm for 6 days (34). After 6 days, the OD_600_ was recorded, and cultures were centrifuged at 7,000 rpm at 4°C for 20 min. Ten milliliters of the supernatant were collected and placed in a 50-mL conical tube, mixed with 30 mL cold 95% ethanol, and placed at −80°C for 30 min and then centrifuged at 7,000 rpm at 4°C for 20 min, and supernatants were discarded. Pellets were washed 2× with 10 mL cold 70% ethanol. A steel grinding ball (size ⅜ in, BC Precision) and 10 mL water were added to the EPS pellet. Then, the tubes were placed inside a shaker (200–300 rpm) for 1 h to break up and resuspend the pellet.

To quantify total EPS, we performed a phenol-sulfuric acid assay to quantify total neutral sugar content. Five hundred microliters of the EPS sample in diH_2_O was added to a clean test tube with a cap. Then, 500 µL 5% phenol (Acros organics, catalog no. 327105000) and 2.5 mL concentrated sulfuric acid (Fisher Scientific, catalog no. S25894) were carefully added to the sample and gently vortexed to mix the solution. Samples were cooled on ice, and the absorbance was then recorded at 488 nm. A dilution series of known concentrations of glucose was used to create a standard curve.

### Cell **surface attachment to glass assay**

This assay was adapted from Espinosa-Urgel et al. (35). Cells were harvested from solid PD3 plates, and the OD_600_ was adjusted to 0.25 in liquid PD3 medium. Five hundred microliters of this was sub-cultured into 5 mL of PD3 in a 13 × 100 mm glass tube. Cultures were incubated at 28°C, 100 rpm for 7 days. Five hundred microliters of 1% crystal violet was added to the culture and allowed to incubate at room temperature for 20 min. The culture and stain mixture was carefully decanted to prevent biofilm disruption. The tube was then gently washed 3× with 1 mL water and allowed to dry overnight. Two milliliters of 30% acetic acid was used to dissolve the crystal violet-stained biofilm. Optical density was measured at OD_600_ using 30% acetic acid as a blank.

### Cell-cell aggregation assay

Cells were harvested from solid PD3 plates and adjusted to OD_600_ of 0.25 in liquid PD3 medium. Five hundred microliters of the culture was subcultured into 5 mL of PD3 in a 13 × 100 mm glass tube. Cultures were incubated at 28°C without agitation for 10 days. After incubation, cultures were gently agitated by hand and then left undisturbed for 20 min to promote settling of cell aggregates to the bottom of the tube. The top 1 mL of the culture was collected (ODs), and its OD_540_ was measured and returned to the culture tube. Then, the entire culture was vortexed thoroughly to disperse cell aggregates, and a 1-mL aliquot was measured at OD_540_ (ODt). The percent aggregation was calculated as [(ODt − ODs)/ODt] * 100.

### Confocal laser scanning microscopy

Cells were harvested from solid PD3 plates, and the OD_600_ was adjusted to 0.25 in liquid PD3 medium. Two hundred microliters of this culture was added to 20 mL of liquid PD3 in a 50-mL conical tube containing a glass slide and incubated at 28°C, 180 rpm. After 4 days, biofilms formed on both sides of the glass slide at the air-liquid interface, the glass slide was removed, and the biofilm on one side of the slide was removed with a Kimwipe. The intact biofilm on the other side of the slide was fixed with 4% paraformaldehyde for 15 min. The fixed biofilm was stained with 20 µM Syto 9 Green Fluorescent Nucleic Acid Stain (Invitrogen, catalog no. S34854) for 15 min in the dark. Slides were gently washed with 1× PBS, and one drop of SlowFade Diamond Antifade Mountant (Invitrogen, catalog no. S36967) was added to the biofilm before placing the cover slip. A Zeiss 880 inverted confocal laser scanning laser microscope (UC Riverside Microscopy and Imaging Core Facility) with an excitation wavelength of 488-nm laser and a 63× oil immersion lens to acquire biofilm z-stack images. A total of five biological replicates were imaged with five images each taken for each biological replicate at random points along the length of the biofilm (beginning from one edge of the slide to the other) for a total of 25 data points.

### BiofilmQ software analysis

Quantitative three-dimensional image analysis of biofilms was performed using BiofilmQ software developed by Hartmann et al. (21). Biofilm z-stack images were denoised by convolution using the default parameters. Floating cells were removed from images, and a threshold of 100 vox was used to remove small artifacts due to noise. The Top-hat filter was set to 15 to remove background fluorescence. Images were segmented automatically using the Otsu algorithm with a sensitivity of 1.75.

### Scanning electron microscopy of *in planta* biofilms

*X. fastidiosa* subsp. *fastidiosa* Temecula1 wild-type and Δ*engXCA2* strains were grown on solid PD3 medium at 28°C for 5 days. Three grapevines were inoculated with 40 µL of *X. fastidiosa* wild-type inoculum in 1× PBS (10^8^ CFU/mL) at the second true internode using the needle-inoculation method described by Hill and Purcell (1). Three grapevines were inoculated with 1× PBS as negative controls. At 15 weeks post inoculation, the internode located 20 nodes above the point of inoculation and the three petioles closest to it were collected, cut into three equal length pieces, and fixed in FAA (70% ethanol, 5% glacial acetic acid, and 5% formalin solution). Samples were prepared for SEM and imaged according to Ingel et al. (36).

### EPS extraction and recombinant EngXCA2 expression for reducing sugar assay

EPS was extracted from Δ*engXCA2* cultures in XFM liquid medium that was slightly modified from Killiny et al. (32) to increase EPS production. In brief, 5 mL hemin chloride (0.05% in NaOH 0.5 µM), 1.5 casamino acids (Acros Organics, catalog no. 61204-1000), 0.25-g bovine serum albumin (Sigma-Aldrich, catalog no. A4503-100G), and 0.1 g galacturonic acid sodium salt (Sigma-Aldrich, catalog no. 73960-5G-F) were added to 200 mL water and filter sterilized (0.22-µm filter). This was then added to 800 mL of autoclaved XFM medium. *X. fastidiosa* was grown, and EPS was extracted as described above. After 6 days of growth, cultures were centrifuged at 7,000 rpm at 4°C for 20 min. Ten milliliters of the supernatant was collected and placed in a 50-mL conical tube, mixed with 30 mL cold 95% ethanol, and stored in a −80°C freezer for 30 min. The solution mixture was centrifuged at 7,000 rpm at 4°C for 20 min, and the supernatant was discarded. The remaining pellet was resuspended in 5 mL water and 500 µL proteinase K (Qiagen, catalog no. 19133) and was incubated at room temperature on an orbital shaker overnight. Following this, the complete EPS extraction protocol described above was repeated, and the resulting pellet was resuspended in 2 mL sodium acetate buffer (100 mM, pH 9). The EPS sample was heated at 95°C for 10 min to deactivate the proteinase K and then dialyzed overnight at 4°C against two changes of 1 L sodium acetate buffer using regenerated cellulose dialysis tubing with a 50-kDa molecular weight cutoff (Spectrum, catalog no. 132542). EPS was quantified using the phenol-sulfuric acid method and stored at −20°C. Recombinant EngXCA2 expression was performed according to Ingel et al. (10).

### Reducing sugar assay

We performed this assay based on the protocol from Gross and Kc (37). In brief, 650 µL of substrate (EPS or carboxymethylcellulose, 0.4 mg/mL) and 650 µL of *Escherichia coli* cell lysate [harboring pMCR7 or pET20b(+) empty vector] were added to a small glass vial and incubated at 37°C, 120 rpm (14, 15). A 200-µL aliquot was removed for each timepoint and added to 1 mL Na_2_[B_4_O_5_(OH_4_)]•8H_2_O and 200 µL 1% 2-cyanoacetamide in a new glass vial. The sample was vortexed and boiled for 10 min. After cooling to room temperature, absorbance was measured at 276 nm using a spectrophotometer. Absorbances were normalized to total protein in lysates from *E. coli* harboring pET20b(+) empty vector or pMCR7 (14, 15). Empty vector absorbance 276 nm/mg protein totals were subtracted from pMCR7. Protein from cell lysates were subjected to SDS-PAGE using a 10% polyacrylamide gel to confirm recombinant EngXCA2 expression. This experiment was performed with three technical replicates and repeated three times.

### Size-exclusion chromatography of exopolysaccharides

The molecular weights of the samples were measured using SEC. For the analysis, lithium chloride (LiCl) and 1,3-dimethyl-2-imidazolidinone (DMI) reagent grade, dextran standards, including 0.76, 1.7, and 3.7 MDa, were utilized. An 8% solution of LiCl/DMI (wt/vol) was prepared by dissolving LiCl in DMI first at room temperature and subsequently heating to 50°C and vortexing. Samples of 2–4 mg of dextran standards, and the analytes (~2.0 mg) were each pretreated by allowing the samples to soak in 1.5-mL nanopure water until dissolved. The suspensions were then freeze dried. The pretreated samples were then dissolved in 8% (~5 mg/mL) LiCl/DMI solution by subjecting the resulting mixture to sequential heating and cooling steps as described by Yanagisawa et al. (38). Sample mixtures were slowly heated to about 80°C and then allowed to slowly cool down. The samples were then diluted to 1% LiCl/DMI solutions with DMI solvent. Of the partially dissolved samples, 0.5 mL was then mixed with 0.5 mL water to fully dissolve. The samples were then frozen and lyophilized overnight to remove as much of the water as possible. The sample solutions were then centrifuged to pellet any remaining undissolved sample material, and the supernatants were carefully collected for SEC.

The method used for SEC of the samples was adapted from Yanagisawa et al. (38). The SEC system used was Agilent 1260 Infinity, which consists of a quaternary pump (G1316A) with a built-in degasser, thermostatted column compartment (G1314A), high-performance autosampler (G1329B), and a refractive index detector (G1362A). An SEC column packed with styrene-divinylbenzene copolymer gel (KD-806M, Shodex, Japan) was used. The eluent was 1% LiCl/DMI solution. SEC conditions were as follows: sample concentration of 0.1%–0.2% (wt/vol), injection volume of 60 µL, flow rate of 0.5 mL/min, and column temperature of 45°C. The RI detector cells were kept at 35°C. Chromatograms of the dextran standards and test samples were collected, and a correlation plot of molecular weight vs elution time was established for each sample.

## Data Availability

All strains used in this study will be made publicly available upon publication and upon request.

## AFTER EPUB

*[This article was published on 13 October 2023 with a grant missing from Acknowledgments. The Acknowledgments were corrected in the current version, posted on 31 October 2023.]*
